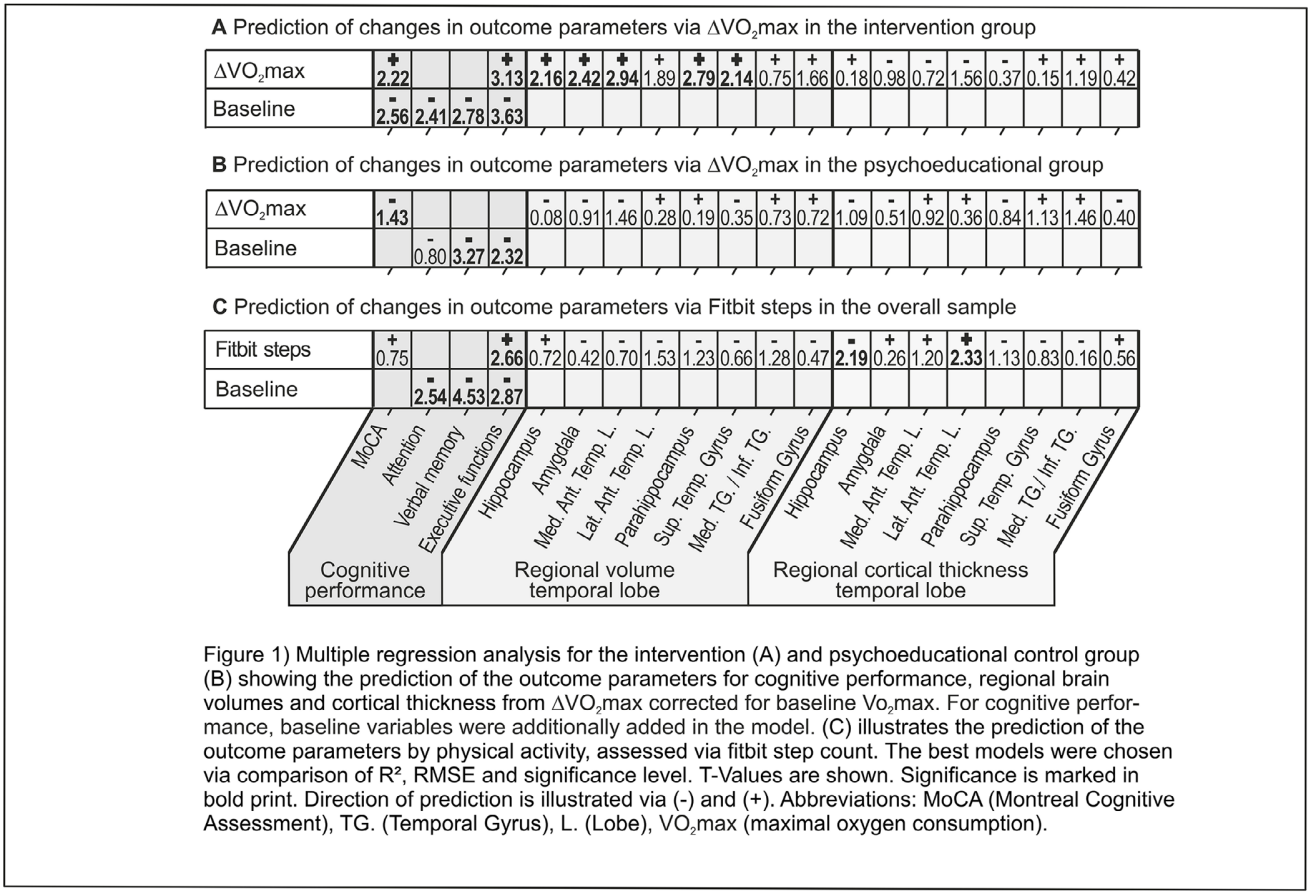# Multimodal Perspective on the Influence of Physical Activity on Alzheimer’s Disease

**DOI:** 10.1002/alz.091460

**Published:** 2025-01-09

**Authors:** Alexa Haeger, Sandro Romanzetti, Christian Hohenfeld, Shari David, Ana Sofia Costa, Luisa Haberl, Frank Hildebrand, Jörg B. Schulz, Kathrin Reetz

**Affiliations:** ^1^ University Hospital RWTH Aachen, Aachen, NRW Germany; ^2^ JARA‐BRAIN Institute Molecular Neuroscience and Neuroimaging, Forschungszentrum Jülich GmbH and RWTH Aachen University, Aachen Germany

## Abstract

**Background:**

Physical exercise presents a viable low‐cost, low‐risk, individual, and widely available non‐pharmacological treatment candidate in cognitive decline such as in Alzheimer’s disease (AD). There are even indications that it can reduce the risk of developing dementia in the first place (Livingston *et al.*, The Lancet, 2020). However, the impact of physical activity and fitness on the multimodal facets of AD, encompassing brain function, cognition and metabolism is still poorly understood.

**Method:**

In the Dementia‐MOVE pilot trial (**M**ulti‐**O**bjective **V**alidation of **E**xercise), 46 patients diagnosed with Alzheimer’s disease were initially included, randomized to either an intervention arm comprising a 6 months‐supervised sports program or a control condition with a psychoeducational program at the RWTH Aachen University Hospital. Participants underwent a comprehensive multimodal assessment of AD relevant parameters, including multimodal MRI, fitness and activity, neuropsychological assessments, blood examinations and the assessments of neuropsychiatric symptoms (Haeger *et al.*, Alzheimers’s & Dementia TRCI, 2020).

**Result:**

In the intervention group, cardiorespiratory fitness change, i.e. ΔVO_2_max, showed a positive association with changes in the Montreal Cognitive Assessment (MoCA), in the executive functions score, and in volumes of the temporal lobe. It also showed a negative correlation with baseline cognitive levels (see also Figure 1 on the multiple regression analysis). High physical activity levels were associated with improved quality of life in the overall sample. In functional MRI using resting state and graph theory (*n* = 20 subjects), we found prediction by ΔVO_2_max for changes on the degree, betweenness, triangles, transitivity, hubness, closeness and Katz centrality, with the last showing the most effects in regions in the temporal lobe. Metabolic MRI comprising ^23^Na‐MRI and ^31^P‐MRS pointed to possible associations of VO_2_max and/or group attribution with changes in brain sodium concentrations/cerebral metabolites.

**Conclusion:**

We show that cardiorespiratory fitness and physical activity examined during an intervention with physical activity can have an impact on different aspects of AD, from cognition, to structural, functional, and metabolic brain alterations. Our results suggest that even patients who are more severely affected by the disease could benefit from interventions in these domains.